# Development and evaluation of a patient education programme for children, adolescents, and young adults with differences of sex development (DSD) and their parents: study protocol of Empower-DSD

**DOI:** 10.1186/s12902-022-01079-3

**Published:** 2022-06-27

**Authors:** Sabine Wiegmann, Martina Ernst, Loretta Ihme, Katja Wechsung, Ute Kalender, Barbara Stöckigt, Annette Richter-Unruh, Sander Vögler, Olaf Hiort, Martina Jürgensen, Louise Marshall, Ingo Menrath, Julia Schneidewind, Isabel Wagner, Julia Rohayem, Klaus-Peter Liesenkötter, Martin Wabitsch, Malaika Fuchs, Gloria Herrmann, Henriette Lutter, Gundula Ernst, Christine Lehmann, Martina Haase, Stephanie Roll, Ralph Schilling, Thomas Keil, Uta Neumann

**Affiliations:** 1 Charité – Universitätsmedizin Berlin, corporate member of Freie Universität Berlin and Humboldt Universität zu Berlin, and Berlin Institut of Health, SPZ Interdisziplinär, Kinderendokrinologie und -diabetologie, Augustenburger Platz 1, 13353 Berlin, Germany; 2 Charité – Universitätsmedizin Berlin, corporate member of Freie Universität Berlin, Humboldt-Universität zu Berlin, and Berlin Institute of Health, Institute of Social Medicine, Epidemiology and Health Economics, Berlin, Germany; 3 Pediatric Endocrinology & Diabetology, St. Josefs Hospital, Ruhr-University Bochum, Bochum, Germany; 4 Division of Paediatric Endocrinology and Diabetes, Department of Paediatrics and Adolescent Medicine, University of Lübeck, Lübeck, Germany; 5 Centre for Reproductive Medicine and Andrology, Clinical and Operative Andrology, University Hospital Münster, Münster, Germany; 6 Endokrinologikum Berlin, Center for Hormonal and Metabolic Disorders, Berlin, Germany; 7 Universitätsklinikum Ulm, Klinik für Kinder- und Jugendmedizin, Sektion Pädiatrische Endokrinologie und Diabetologie, Hormonzentrum für Kinder und Jugendliche, Ulm, Germany; 8 Department of Medical Psychology, Hannover Medical School, Hannover, Germany; 9 Förderkreis Schulung chronisch kranker Kinder und Jugendlicher e.V., Berlin, Germany; 10 Institute of Clinical Epidemiology and Biometry, Würzburg University, Würzburg, Germany; 11 State Institute of Health, Bavarian Health and Food Safety Authority, Erlangen, Germany

**Keywords:** Empowerment, Patient education, DSD, Differences of sexual development, Modular education, Children, Adolescents, Parents, Training

## Abstract

**Background:**

Differences in sexual development (DSD) are rare diseases, which affect the chromosomal, anatomical or gonadal sex differentiation. Although patient education is recommended as essential in a holistic care approach, standardised programmes are still lacking. The present protocol describes the aims, study design and methods of the Empower-DSD project, which developed an age-adapted multidisciplinary education programme to improve the diagnosis-specific knowledge, skills and empowerment of patients and their parents.

**Methods:**

The new patient education programme was developed for children, adolescents and young adults with congenital adrenal hyperplasia, Turner syndrome, Klinefelter syndrome or XX-/or XY-DSD and their parents. The quantitative and qualitative evaluation methods include standardised questionnaires, semi-structured interviews, and participatory observation. The main outcomes (assessed three and six months after the end of the programme) are health-related quality of life, disease burden, coping, and diagnosis-specific knowledge. The qualitative evaluation examines individual expectations and perceptions of the programme. The results of the quantitative and qualitative evaluation will be triangulated.

**Discussion:**

The study Empower-DSD was designed to reduce knowledge gaps regarding the feasibility, acceptance and effects of standardised patient education programmes for children and youth with DSD and their parents. A modular structured patient education programme with four generic and three diagnosis-specific modules based on the ModuS concept previously established for other chronic diseases was developed. The topics, learning objectives and recommended teaching methods are summarised in the structured curricula, one for each diagnosis and age group. At five study centres, 56 trainers were qualified for the implementation of the training programmes. A total of 336 subjects have been already enrolled in the study. The recruitment will go on until August 2022, the last follow-up survey is scheduled for February 2023. The results will help improve multidisciplinary and integrated care for children and youth with DSD and their families.

**Trial registration:**

German Clinical Trials Register, DRKS00023096. Registered 8 October 2020 – Retrospectively registered.

## Background

Differences of sexual development (DSD) are rare conditions with atypical development of chromosomal, gonadal, or anatomic sex [1, 2]. The diagnoses are classified into sex chromosome DSD (e.g. Turner syndrome, Klinefelter syndrome or 46,XX/46,XY chimerism), 46,XY DSD (e.g. disorders of gonadal development or disorders of androgen synthesis or action) and 46,XX DSD (e.g. disorders of gonadal development or androgen excess) [3]. Sexual development begins during fetal life and continues in childhood and adolescence. It is one of the most personal and private areas of life and discussing sexual themes is often combined with shame and anxiety [4]. It was the hard work and experiences of individuals with DSD, which has changed the medical care over the last decades. Open communication with the individuals with DSD and their families is the basis of care and participation in decision making is encouraged [2, 4]. With this approach, the decisional regret is minimised due to satisfaction with the decision making process [5]. To follow that process, individuals with DSD and their families have to be comprehensively informed. Therefore, the care providers should decrease the complexity of their communication [6], which is challenging in some variances of sexual development. The carers should also include all topics which are important for the individuals and families. Every stage of life has its own challenges. While at birth, for example, questions about gender assignment, naming or the communication with family members are important. Later in childhood or adolescence, there are questions about dealing with the close social environment, day care and school as well as questions about fertility. At any age, worries about the child’s social adaptation and adjustment are of concern combined with the fear of insensitive reactions and social stigmatisation [7].

Initial studies have demonstrated that adults with DSD and their parents present a reduced quality of life (QoL) [8–10]. Learning coping skills and communication strategies can improve parents´ psychosocial adaptation [11, 12]. Even in childhood and adolescence, psychological disorders, decreased QoL, self-esteem, and school abilities have been described [13, 14]. In recent years, various international DSD experts have recommended the need for psychosocial care from childhood to adulthood [7, 15, 16]. One important aspect within the care and education of children with DSD is the parent–child sexual communication to convey knowledge as well as to have discussion regarding anatomy, sex, values, beliefs, and expectations [4, 17, 18].

However, standardised approaches for training and psychosocial support of DSD patients are missing so far. Previous research has demonstrated that patient education programmes do increase the health- and disease-related knowledge, quality of life and thus reduce disease-specific burden [19]. It was shown in MRKH syndrome that group psychological interventions can improve well-being [20]. Patient education programmes are standardised, manualised, interactive group programmes for patients with chronic diseases [21]. In recent years, the importance of patient education programmes for chronic diseases has been recognised and training programmes have been developed for numerous conditions [22–25]. They focus on self-management, empowerment, psychosocial support and disease-specific knowledge and skills [26].

An already existing structured and established training programmes is ModuS, a modular group patient education approach. It addresses children and adolescents with a chronic condition and their families. This approach consists of a standardised modular structure that includes four generic modules and three diagnosis-specific modules [27]. The generic modules are applied in all education programmes on the assumption that all chronic conditions in childhood have similar psychosocial issues. The diagnosis-specific modules cover the medical topics relevant to the specific diagnosis [19, 27].

So far, no interdisciplinary structured patient education programme exists for children and youth with DSD and their parents. For this reason, the Empower-DSD project was initiated to develop, conduct and evaluate a modular group education programme for children and youth with congenital adrenal hyperplasia (CAH), Turner syndrome, Klinefelter syndrome, and XX-/or XY-DSD including the Mayer-Rokitansky-Küster-Hauser syndrome (MRKH), and their parents to improve the quality of life, the diagnosis-specific knowledge, and empowerment of patients and their parents. The present protocol describes the aims, study design and methods of the newly developed age-adapted multidisciplinary education programme.

## Methods/Design

### Study design

Empower-DSD is a prospective longitudinal, mixed methods, non-controlled multicentre study. The project is funded by the innovation fund by the German Federal Joint Committee (01VSF18022), which was established to improve Health Services Research and to develop new health care models in order to integrate them after positive evaluation into routine care. One of the objectives of the study is to develop, implement and evaluate an age-adapted education programme for children and youth with DSD. A second aim of the Empower-DSD study is to develop and evaluate an information management concept for families newly diagnosed with DSD. The methods and results of this project part are outside the scope of the current description.

The primary outcome is the health-related quality of life of participants of the education programme. As secondary outcomes, the diagnosis-specific knowledge, coping, disease burden, and shame about the condition will be observed. Overall, satisfaction with the education programme will be qualitatively evaluated. The qualitative study parts examine individual expectations and perceptions of the programme.

### Partners

**Fig. 1:**
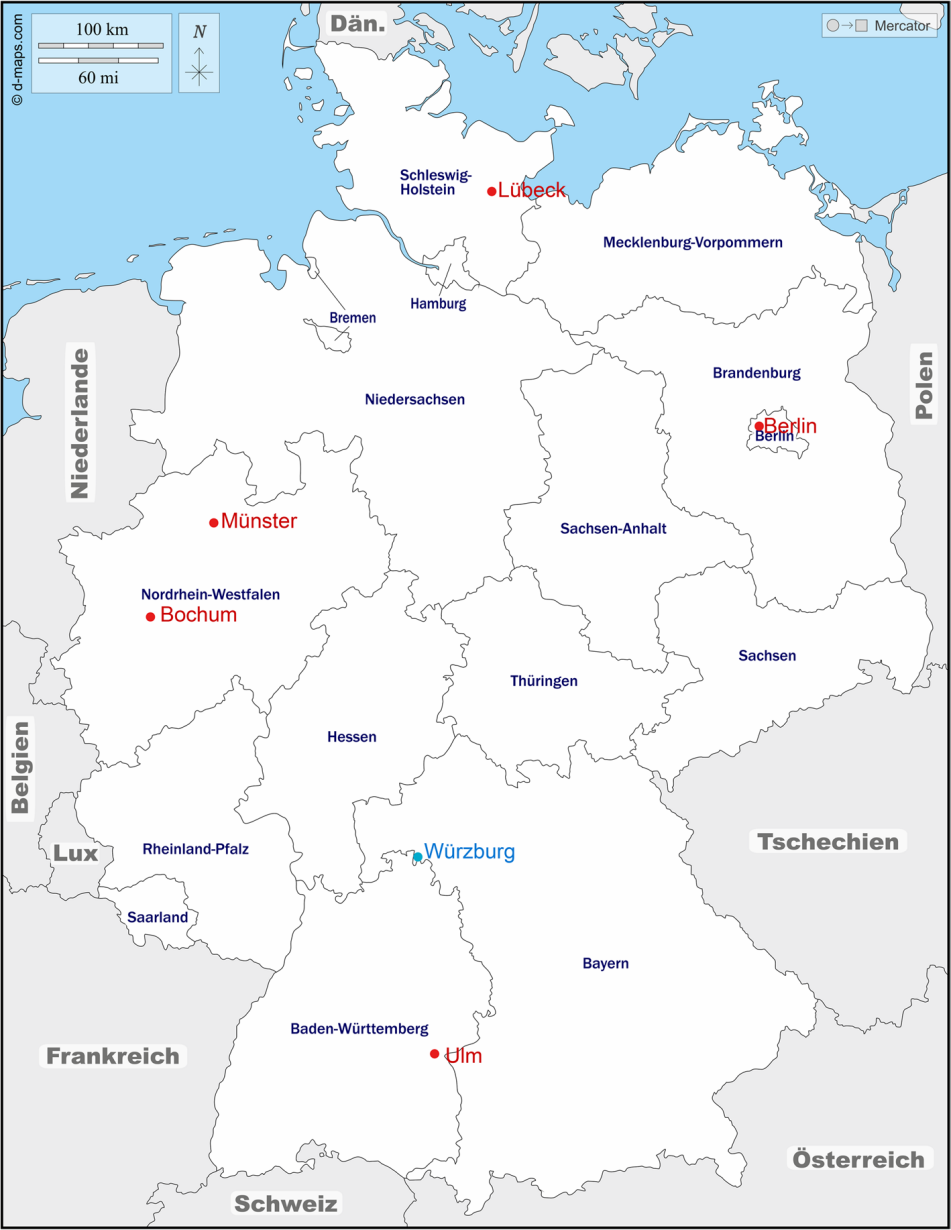
Empower-DSD consortium ( source: https://d-maps.com/carte.php?num_car=4692&lang=de).  Central data management.  University hospitals with DSD study centres

The Empower-DSD study group consists of five university hospitals across Germany (Lübeck, Berlin, Münster, Bochum, and Ulm) with specialised departments for the care of children and young adults with DSD. An additional partner, the University of Würzburg (Institute of Clinical Epidemiology and Biometry) is responsible for the central data management and data quality assurance as well as the development of web-based questionnaires using REDCap (Fig. 1).

In order to address the patient’s perspective in the development of the training programme and the evaluation approach, the study group works in close cooperation with German patient support groups for CAH (AGS-Eltern- und Patienteninitiative e.V.), XX-/XY-DSD (Intergeschlechtliche Menschen e.V. (IMeV) and SHG Interfamilien), Klinefelter syndrome (47xxy klinefelter syndrom e.V.), and Turner syndrome (Turner-Syndrom-Vereinigung Deutschland e.V.).

### Development of the patient education programme

**Fig. 2:**
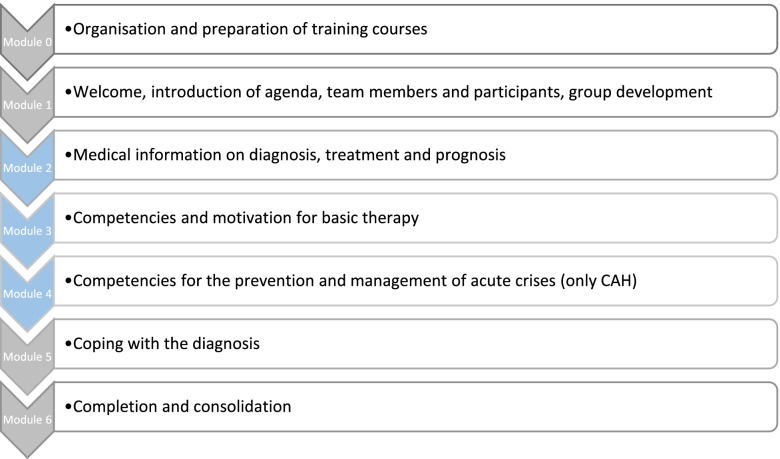
Modules of ModuS education programme for DSD diagnoses, according to Ernst et al. [27].  generic modules.  diagnosis-specific modules

In a first step, the DSD study group developed a curricular framework based on the ModuS concept, which was previously established by Ernst and colleagues for children with chronic diseases and their parents [27]. The standardised modular structure was adapted to the DSD diagnoses with four generic and three diagnosis-specific modules. A diagnosis-specific module that covers behaviour in acute illness was included in the CAH curriculum only, since diagnosis-related medical emergencies do not occur in the other DSD groups (Fig. 2). The modular approach allows adding or omitting topics that are relevant to the target group. A generic transition module which was developed by ModuS was not included [28], but relevant topics and learning objectives were integrated in the curriculum for adolescents.

**Fig. 3:**
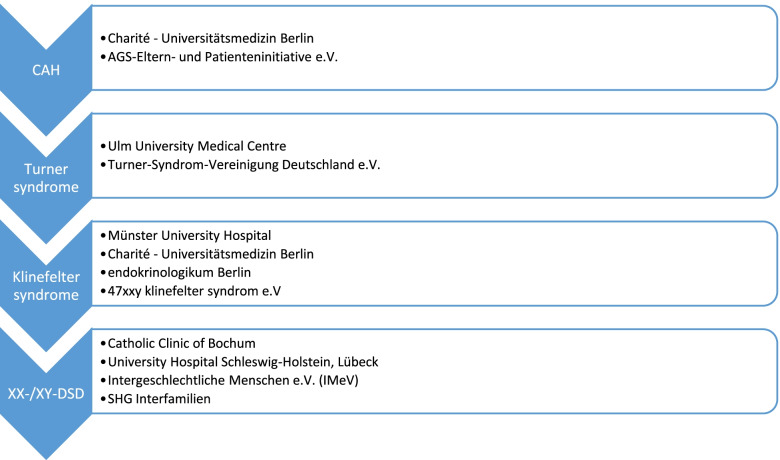
Working groups for diagnosis-specific modules

In the next step, diagnosis-specific working groups consisting of medical and psychological staff members of the study centres and representatives of the patient support groups were formed (Fig. 3). Those working groups collected relevant topics and arranged them into the modular structure. Based on the aims of the project and the module topics, learning objectives were developed separately for children, youth and parents or relatives. In a circular, participative, and communicative process, the learning objectives were discussed and approved by all members of the working group. In a meeting of the entire study group, the topics and related learning objectives of all diagnosis-specific curricula were presented, discussed and consented.

Further, the working groups developed ideas for the teaching methods and materials used to achieve the learning objectives (e.g. worksheets, models, pictures). The study group aimed for an inclusive language in all documents. A working group therefore prepared a list of terms to use and terms to avoid in the curricula and teaching materials (Tab. 1). As an example, there is still an ongoing discussion about the term DSD; as it could be read as *differences* of sexual development or *disorders* of sexual development. We agreed in the Empower-DSD study group to use the term *differences* of sexual development. Additionally, the questionnaires of the quantitative analysis and the interview guide for the qualitative analysis were explicitly adapted to the agreements on an inclusive language for DSD.

**Table 1:** Exemplary examples for an inclusive language in Empower-DSD

Terms to use	Terms to avoid
variation of genital development	ambiguous/ atypical genitalia
child/ person with DSD	affected child/ person
differences of sexual development	disorders of sexual development

### Training academy

To ensure the quality and reproducibility of the education programme, all professionals have to complete a training academy of a basic training and an advanced diagnosis-specific training that is an integral part of the ModuS concept. The basic training academy was developed by the “Kompetenznetz Patientenschulung e.V.” and was provided for participants of different specialties by the “Förderkreis Schulung chronisch kranker Kinder und Jugendlicher e.V.”. It includes general information on the organisation of a patient education programme, the role and tasks of the trainer, and general information on the implementation of the ModuS concept. The advanced DSD-specific training academy is based on the developed DSD education programme and provides knowledge about medical content of the programme, as well as specific teaching methods and the use of an inclusive language for the DSD education programme.

### Sample and recruitment

The modular education programme is conducted for children and young adults aged 6–24 years with DSD and their parents, as well as for families with a child newly diagnosed with DSD within the last two years. The potential study participants will be contacted by the study centres and their cooperating institutions, such as regional endocrinologists, patient support groups or other hospitals. The recruitment includes e-mail or phone contact after initial information by the physician during the consultation, information by a project flyer distributed by one of the partners or information from the Empower-DSD website (https://empower-dsd.charite.de). Additionally, professionals and peers who work with or consult patients with DSD and their families and who are involved in the development of the Empower-DSD concepts are recruited for the qualitative evaluation of the study. The inclusion criteria are presented in Table 2. Subjects are excluded if informed consent is missing, if they are participating in another study addressing patient education related to DSD or if a language barrier disables participants from taking part in the programme.

**Table 2:** Inclusion criteria

Inclusion criteria
Patients with congenital adrenal hyperplasia (CAH), Klinefelter syndrome, Turner syndrome, XX-/XY-DSD including MRKH
Confirmation of diagnosis by chromosomal analysis, genetic test result, laboratory test or clinical examination
Children between 8–13 years (according to their cognitive development from the age of 6 years)
adolescents/young adults between 14–24 years
Parents/ caregivers of children or adolescents with DSD newly diagnosed in the last two years
Professionals who provide the patient education programme or have been involved in its development
Peers who participate in the patient education programme as peer counsellors or were involved in its development process
Written informed consent is available from patients (6 years and older), parents/ caregivers, peers, and professionals

### Sample size

The study follows an explorative approach and aims to include as many cases as possible in all five centres during the field phase. A sample size of 300 participants provides a power of over 95% with moderate effects of the education programme (standardised difference of paired means of 0.4) with an exploratory significance level of 0.05 (two-sided). Due to an expected drop-out of less than 15%, the aim is to recruit 350 children and 350 adolescents/young adults for the study. This lost-to-follow-up is considered realistic from previous paediatric research projects, because the subjects in the current study will have frequent consultations with the team of the department of endocrinology due to their diagnosis. Approximately 160 *newly* diagnosed cases are expected to occur in all centres together during the observation period based on previous year’s occurrence. Therefore, a participation of 100 families with newly diagnosed children in the study seems realistic. To ensure the participation of the families until the last follow-up, a strict reminder management is carried out and gift cards are awarded among all participants who have completed all questionnaires.

Regarding the qualitative evaluation, we plan to conduct approximately 50 semi-structured interviews with patients, parents, professionals, and peers taking part or working in the education programme. Based on the explorative assumptions of qualitative research, the number or content of the interviews can be adjusted until theoretical saturation is reached.

### Evaluation

The education programme will be quantitatively and qualitatively evaluated by means of standardised questionnaires, interviews and participating observations. For the quantitative evaluation, patients and their relatives who participate in the education programme are requested to fulfil online questionnaires at four time points: at baseline before participation in the education programme (t0), immediately after participation in the education programme (t1), and at a follow-up 3 months (t2) and 6 months (t3) after participation. Table 3 shows the questionnaires used at the different time points in the evaluation.

**Table 3:** Questionnaires of the quantitative evaluation of the study

Subgroup of sample	Time point	Standardised questionnaires	Self-constructed items
Parents of children under 6 years (includes parents of newly diagnosed children under 6 years)	T0, T2, T3	Cantril ladder, WHO-5	Sociodemographic data, diagnosis-specific items, social support, gender, maleness and femaleness, shame, self-esteem, disease burden and general coping, knowledge
Parents of children older than 6 years (includes parents of newly diagnosed children older than 6 years)	T0, T2, T3	Cantril ladder, KINDL-R parents, WHO-5	Sociodemographic data, diagnosis-specific items, social support, gender, maleness and femaleness, shame, self-esteem, disease burden and general coping, knowledge
Children 6–13 years	T0, T2, T3	Cantril ladder, KINDL-R (version 7–13 years), CODI (for children older than 7 years), BIS (for children older than 12 years)	Sociodemographic data, diagnosis-specific items, social support, gender, maleness and femaleness, shame, self-esteem, disease burden and general coping, knowledge
Adolescents 14–17 years	T0, T2, T3	Cantril ladder, KINDL-R (version 14–17 years), CODI, BIS	Sociodemographic data, diagnosis-specific items, social support, gender, maleness and femaleness, shame, self-esteem, disease burden and general coping, knowledge
Young adults 18–14 years	T0, T2, T3	Cantril ladder, WHO-5, BIS	Sociodemographic data, diagnosis-specific items, social support, gender, maleness and femaleness, shame, self-esteem, disease burden and general coping, knowledge
Children and adolescents 6–17 years	T1		satisfaction with programme, short version
Young adults 18–14 years	T1	Adapted ZUF-8	satisfaction with education
Parents of all children and adolescents	T1	Adapted ZUF-8	satisfaction with education

T0 before participation in the patient education programme, T1 immediately after participation in the education programme, T2 3 month after participation, T3 6 month after participation

For the qualitative evaluation of the education programme, semi-structured interviews and participatory observation are conducted continuously during the implementation process with patients and their families, professionals and peers. The key themes covered through the interviews are expectations and wishes regarding the education programme, experiences and satisfaction with the education programme, impact of the education programme on life satisfaction and dealing with the diagnosis, and for professionals and peers the impact of the programme on their work and work satisfaction.

Interviews with children and adolescents provide limited information about their experiences, thoughts and perspectives [29], especially within telephone interviews due to the current pandemic situation [30]. Participating observations can underline and enhance the statements made in the interviews or replace interviews and are therefore added to the qualitative study design.

Finally, to extend and deepen the findings, the quantitative and qualitative results will be related to each other (triangulation).

### Instruments

The online assessment contains standardised questionnaires and self-constructed questions, which are described in the following.

#### Sociodemographic data

Sociodemographic data such as age, nationality, education, and general questions about the diagnosis are asked.

#### Health-related quality of life

The health-related quality of life (HRQoL) is the primary outcome of this study and is assessed by the KINDL-R. This is a generic instrument to assess HRQoL in children between the ages of 4 and 17 years [31]. The instrument includes the domains of physical and psychological well-being, social relationships with family and friends, self-esteem and everyday life activities. Moreover, the assessment consists of age-specific questionnaires for children and adolescents as well as questionnaires for parents about their child. In the present study, the versions for children aged 7–13 years and 14–17 years are used, as well as the corresponding questionnaires for parents. Studies revealed a sufficient reliability (Cronbach’s alpha up to 0.80) and validity (*r* > 0.60) in samples of healthy or chronically ill children [31]. The tool was already used in a study population of children with differences of sexual development [14] and normative data exist for the population of children and adolescents in Germany in general [32].

#### Life satisfaction and well-being

To assess general life satisfaction and well-being, the Cantril ladder and the WHO-5 are used. The Cantril ladder is a visual scale on which subjective life satisfaction is marked on ladder levels between 0 and 10 [33]. The highest level represents the best possible life and the lowest level the worst possible life. The respondent should mark the personal life satisfaction of the present moment. The original version of the Cantril ladder was developed for the use in adult populations [34, 35]. The reliability and reproducibility have been confirmed for several life satisfaction scales. They reflect different living conditions and make a valid statement regarding future behaviour [36]. For samples with children and adolescents, an adapted version without an initial imagination of the best and worst life has been developed for the Health Behaviour in School-aged Children (HBSC) studies [37]. Good validity and reliability for this adapted version has been confirmed by several studies [33, 34]. In the present study, the adapted version for children, adolescents, young adults as well as for parents is used to reflect on one's own life satisfaction.

The WHO-5 instrument is a generic assessment of psychological well-being, which is widely used all over the world and translated into 31 languages. Psychological well-being is an essential dimension of HRQoL [38]. The WHO-5 has a good psychological and clinical validity across different settings and samples. In the context of chronic diseases and diagnostic screening, it is even able to reveal depression [39]. In this study, the instrument is used for the adult sample, including parents and patients older than 18 years as a main outcome measure.

#### Disease burden and coping

Disease burden and coping are assessed by the CODI (**co**ping with a **di**sease) questionnaire and self-constructed questions. The CODI is a 28-items questionnaire that focusses on coping strategies of children and adolescents between 8 and 18 years in the context of chronic diseases [40]. The items cover the coping strategies acceptance, avoidance, cognition, distance, emotional reaction, wishful thinking, and general coping. Due to the focus on disease-specific coping strategies and the use of pathology-oriented language, the items were modified with regard to an inclusive language. In all items containing the word "disease", the word was replaced by "diagnosis" to reflect the understanding and attitude towards DSD as a variation rather than a pathology of genital development. This instrument has been already used in a sample of young patients with DSD, where the word “disease” was replaced by a blank gap to fill in a personal word describing one’s diagnosis [41]. Reference data are available from the German Health Survey for Children and Adolescents (KiGGS) [42] and other studies using the instrument [43, 44]. The reliability of the questionnaire has a Cronbach's alpha between 0.72 and 0.88 [42].

In addition, the questionnaire contains self-constructed questions about the social support, disease burden and general coping. The items to assess the rate of burden are orientated at the assessment of Mueller-Godeffroy et al. [45]. Two questions out of the 4-item-assessment are used to evaluate the general burden of the parents and their perceptions of their own child. Two other questions evaluate the supportive and social network and the use of patient support groups.

#### Body satisfaction and self-perception

To assess attitudes and perceptions of gender and body among individuals with DSD, no published standardised instrument was appropriate for our research questions. Thus, twelve items were developed to evaluate the own satisfaction or perceived satisfaction of the child’s body, gender, and maleness and femaleness, respectively. The responses can be chosen between very satisfied and very dissatisfied and ‘no answer possible’. For the questions about maleness and femaleness, the respondents can classify on a 5-point Likert scale between very male and very female, ‘sometimes like this, sometimes like that’ or ‘I don’t know’. Self-esteem and shame are asked in three questions about the persons and the feelings associated with talking about the diagnosis and the body.

The Body Image Scale (BIS) is used to evaluate the satisfaction with body parts like primary gender, secondary gender, and hormonally unresponsive attributes [46]. It comprises 30 body features that the respondent is asked to rate on a 5-point Likert scale between very satisfied and very dissatisfied. In the original version, the respondent was asked about the desire to change a body part by surgery, but this was beyond the scope of the present study and has therefore been omitted [46]. The instrument showed a consistent reproducibility of the scores [46]. The outcome measure is used in a German translation, that was already applied to a German sample of adolescents with DSD [41]. In the present study, the BIS is used for all participants between 12 and 24 years.

#### Diagnosis-specific knowledge

To evaluate the improvement of diagnosis-specific knowledge, self-constructed multiple-choice items were developed. The questionnaire comprises six items for each diagnosis, for both children and parents, but with different perspectives and levels of difficulty. The same questions are asked before and then at follow-up after three and six month after the education programme. For statistical evaluation, the individual items are added up to an overall score.

#### Satisfaction with programme

The questionnaire for the evaluation of the satisfaction with the education programme is based on an instrument, which was originally developed for the assessment of patient satisfaction with inpatient care [47]. Psychometric properties were evaluated as high, with a Cronbach’s alpha between 0.88 and 0.92 [48]. For programme evaluation, the items were adapted and further questions were added. A similar adapted version was already used in the evaluation of an asthma-specific ModuS programme [27]. Basic quality requirements of patient education programmes were discussed and transformed into items of the questionnaire [49]. Finally, ten questions with a 4-point Likert scale and two open-ended questions are set for the survey immediately after the training for parents and adults. To evaluate children’s and adolescents’ satisfaction, a shorter version with one item on general satisfaction, two open-ended questions, and four items with a 5-point smiley scale is used.

### Data management

For the online-based data assessment we use the remote data capture system REDCap. Separate questionnaires were programmed for the target groups based on age and diagnostic groups. Before the study centres started the field work, they were trained in using REDCap by the central data management following a standardised training scheme.

The participant information form contains all the relevant information used to assign the diagnosis- and target group-specific questionnaires in the surveys. Family relationships are also assigned via this form, so that the responses of the parents can be linked to those of the associated children. The study included the following 4 groups: (i) children aged 6 to 13 years, (ii) adolescents aged 14 to 17 years, (iii) young adults aged 18 to 24 years (all groups with links to the corresponding parental questionnaires), (iv) parents of children newly diagnosed within the last two years. Baseline and follow-up assessments were separately programmed for each age group. Access codes for baseline and 3 follow-up assessments were generated for each participant and included the individual participant information.

Complex alpha-numeric pseudonyms are used as links between diagnosis- and age group-specific questionnaires. The pseudonyms were generated at the central data management. A list with a sufficient number of pseudonyms together with guidelines for documenting the use of them was sent to each study centre before the recruitment started.

The following quality assurance tasks are important in this project phase on the part of the central data management:

(i) Checking the family assignments for correctness, if necessary submit queries to the study centre. This ensures the correct assignment of questionnaires to the different assessment time points, but it is also essential for future evaluations in terms of linking questionnaire responses of parents with those of their children.

(ii) Verifying that questionnaires have been completed. A message regarding the completeness of the questionnaires is also included in the participant information. This was subsequently added by the central data management to help the local study nurses keeping track.

During the course of the project, it is important to keep a close communication between the central data management and the study centres to identify sources of error early and to correct them quickly, if possible.

Personal data from the qualitative interviews (e.g. audios) are only stored digitally with special access authorisation. Audios, complete transcripts or other person-identifying data are only transport encrypted, e.g., with a VeraCrypt container. Only pseudonymised data will be analysed.

### Statistical methods

Descriptive statistics will be presented for the subsamples children (6–13 years), adolescents (14–17 years), young adults (18–24 years), and parents separately. Categorical variables will be reported as absolute and relative frequencies (with 95% confidence interval), normal distributed metric variables as mean and standard deviation, and skewed variables as median with interquartile range.

Pre-post comparisons (before vs. after training) will be conducted with statistical tests for paired samples (single-factor ANOVA with repeated measures, one-sample t-test, McNemar test) separately for children, adolescents, young adults, and parents. All tests are two-sided with a significance level of 0.05. However, *p*-values are considered exploratory, without adjustment for multiple testing. Missing data will not be imputed. No interim analyses are planned. A statistical analysis plan will be prepared prior to data analysis.

### Qualitative evaluation

For the qualitative evaluation, two experienced researchers will perform semi-structured interviews and participatory observation. Preferably, interviews are conducted in a face-to-face situation, but can also be carried out as a telephone or video interview if required. All interviews will be recorded digitally, pseudonymised, and transcribed verbatim. Further data material contains the written memos and logbooks of the research process, interview protocols and observational protocols. These can add further information on the setting or nonverbal expressions of the interviewed or observed subjects. For the analysis, the transcripts are imported into the computer programme MAXQDA®. Data analysis is carried out by developing categories inductively from the material and deductively from the key themes of the interview guide. The analytic process will be circular, meaning that new insights from the interviews and initial data analysis will be included into possible revision of the interview guide and subsequent data interpretation. The analysis will be discussed in regular interdisciplinary team meetings to enhance intersubjectivity and multiperspectivity.

### Triangulation

In a further analysis step, the quantitative and qualitative study results will be triangulated, i.e., related to each other. This will allow to supplement, extend, and deepen the findings of the study, and will generate further hypotheses about the possible effects and experiences. The mixed-methods approach of this study follows a parallel design (quantitative and qualitative data are collected in parallel). The integration of quantitative and qualitative data is data- and outcome-based [50].

### Ethics

The study design follows the principles of the Declaration of Helsinki. The local ethics committee at the leading study centre Charité—Universitätsmedizin Berlin approved the study protocol, procedures, and consent forms (EA2/238/19). All participating study centres obtained ethical approval by their institutions.

## Discussion

Empower-DSD is a multicentre study to develop and evaluate an education programme for children, adolescents and young adults with DSD and their parents. This comprehensive education programme based on the ModuS concept [27] contains medical information as well as psychological issues arising from the diagnosis and is taught in an age-appropriate manner. From May 2020 to August 2022, the age- and diagnosis-specific education programme for CAH, Turner syndrome, Klinefelter syndrome and XX- or XY-DSD are provided at five study centres in Germany and will be evaluated quantitatively and qualitatively. This paper reports on the curriculum development process, the study design, and the evaluation concept.

As a first result of the Empower-DSD study, a specific DSD patient education programme is developed. For each diagnosis and for each age group (children, adolescents and young adults, parents/carers), a separate curriculum specifies learning objectives, content, and learning methods. Each manual includes an exemplary schedule for the implementation of the education programme. According to the manuals, the advanced DSD training academy was established and a total of 56 professionals, e.g., medical doctors, psychologists, social workers or medical assistants, were qualified to conduct the educational programme in the different study centres. The education programme is offered in groups separated by age and diagnosis. Usually, an education course takes two days with a total of 12–14 lessons of 45 min each. The courses of the children/adolescents take place simultaneously with the training of parents/carers. The groups are limited to 4–8 families. Furthermore, peer counsellors are an integral part of the educational programme. At each date, a peer from the cooperating patient support group is available to talk about the peer’s own experience in daily life with the diagnosis and to answer questions. Up to this day, a total of 336 subjects have been enrolled in the study and took part in the educational programme. Meanwhile, there have already been educational events provided for each diagnosis at least once. After each event, a feedback discussion takes place and the trainers of a centre fulfil a structured feedback form including information on organisation, methods, positive and negative training experiences to improve the quality of the educational programme. In order to benefit from the experiences of the other study centres, regular online meetings are held between the trainers.

The modular patient education approach (ModuS) was developed for rare chronic diseases in childhood. It was shown that this programme has positive effects on quality of life, knowledge and disease burden [19, 27]. For that reason, this ModuS concept served as a base for the curriculum of the DSD education programme. The modular structure allowed an adaptation to the needs of the different diagnoses CAH, Turner syndrome, Klinefelter syndrome, and XX- or XY-DSD including MRKH. Although there is a manual for every diagnosis, there are different themes which were developed once and could be used across all diagnoses, e.g., the explanations of chromosomes, hormones, pubertal development and others. On the other hand there are specific themes which are important for only one diagnose, e.g., the whole module 4 including competencies for the prevention and management of acute crises which was only included in the CAH programme. Next to the medical information, the psychosocial aspects are a main part of the programme under the assumption that psychosocial issues are at least equally as important as medical issues [19]. In the care of people with DSD, psychosocial care is obviously needed and should be an integral part of management [51, 52]. The psychological learning objectives were developed across all diagnoses, but have to be modulated to special needs for every single diagnosis.

All modules have been adapted for the inclusive language and for special issues comprising the DSD diagnoses. Finding a common language was a major challenge. Thus, modifications had to be made in terms of the inclusive language used, e.g., the word “diagnosis” is used instead of “disease” or the word “difference” instead of “disorder”. Here, the involvement of the patient support groups in the development of the trainings was a very valuable process [53, 54]. Some of the patient support groups had difficulties with embedding the education programme in a medical system, while others did not. The pathologising of their difference of sexual development as a “disease” was seen critically by many people with DSD. Since they do not consider themselves as being ill, they feel that they have no need for training in a hospital setting. Due to the rarity of the diagnoses, this is also a challenge in terms of recruitment. In a constructive and discursive process with the patient support groups, an attempt was to integrate all these perspectives in order to develop the best possible programme for the participants. These discussions can also be found in creating the evaluation contents. The development of the interview guides for the qualitative study part were circulated in the whole study team including the support groups and discussed intensively. The same process took place in the development of quantitative questions, e.g., about diagnosis-specific knowledge. This approach was intended to ensure an education programme with comprehensive, age-appropriate information and also information relevant for everyday life for the participants. The training of children, young people and parents in different groups addresses the different needs in knowledge transfer. Particularly for adolescents and young adults, special emphasis was placed on the examinations that will also be necessary later in life in order to improve the transition to adult medicine. In case of a necessary hormone replacement therapy, adherence can be improved by providing good and comprehensive information on the effects and side-effects of medical treatments [55].

In addition, in terms of learning methods, the needs of the different age groups had to be considered. Children and juveniles need a more playful approach to the content, while parents benefit from informative presentations and the group discussions with other families and experts [27]. For youth, learning objectives related to transition are an important element in increasing transition skills, self-efficacy, and satisfaction with school [56]. In general, the social contact and informal exchange are at least as important as the lessons on medical and psychological topics. The educational programme offers a protected setting to address individual concerns with psychological experts and peers with the aim to reduce diagnosis-specific burden and to improve the HRQoL [19]. Empowerment and self-management as main objectives of the ModuS concept are also taken into account in the DSD curriculum [27]. Due to the Sars-CoV-2-pandemic, we had to continuously reflect on whether these educations are also possible as online events, because the restrictions did not allow educations to be held in person for several months. However, the strength of the education programme is the interactive and communicative approach, which allows the participants to share their very sensitive and personal issues, which is probably not possible in an online course. So, we are looking forward to conducting further trainings in presence at our five study centres. In addition to the scientific evaluation, there is continuous reflection on the trainings, and at the end of the study the results will be incorporated into the revision of the curriculum.

The present protocol describes the aims, study design and methods of a newly developed age-specific multidisciplinary DSD education programme to improve the diagnosis-specific knowledge, skills and empowerment of children, youth and young adults with DSD and their parents. Empower-DSD will reduce knowledge gaps regarding the feasibility, acceptance and effects of standardised patient education programmes and will help to develop better multidisciplinary and integrated treatment strategies of high quality for children and young adults with DSD and their families.

## Data Availability

The datasets generated and analysed during the current study are available from the corresponding author on reasonable request. A specific research objective must be presented by an exposé.

## Abbreviations

CAH: Congenital adrenal hyperplasia
CODI: Coping with a disease
DSD: Differences of sexual development
HBSC: Health Behaviour in School-aged Children study
HRQoL: Health-related quality of life
ModuS: Modulares Schulungsprogramm (Modular education program for chronic diseases in childhood)
MRKH: Mayer-Rokitansky-Küster-Hauser syndrome
QoL: Quality of life
RedCap: Research Electronic Data Capture
WHO: World Health Organisation
